# A quality metric for homology modeling: the H-factor

**DOI:** 10.1186/1471-2105-12-48

**Published:** 2011-02-04

**Authors:** Eric di Luccio, Patrice Koehl

**Affiliations:** 1 Computer Science Department, Room 4337, Genome Center, GBSF University of California Davis 451 East Health Sciences Drive Davis, CA 95616, USA; 2 School of Applied Biosciences, Kyungpook National University (KNU), 1370 Sangyeok-dong, Buk-gu, Daegu, 702-701, Republic of Korea

## Abstract

**Background:**

The analysis of protein structures provides fundamental insight into most biochemical functions and consequently into the cause and possible treatment of diseases. As the structures of most known proteins cannot be solved experimentally for technical or sometimes simply for time constraints, *in silico *protein structure prediction is expected to step in and generate a more complete picture of the protein structure universe. Molecular modeling of protein structures is a fast growing field and tremendous works have been done since the publication of the very first model. The growth of modeling techniques and more specifically of those that rely on the existing experimental knowledge of protein structures is intimately linked to the developments of high resolution, experimental techniques such as NMR, X-ray crystallography and electron microscopy. This strong connection between experimental and *in silico *methods is however not devoid of criticisms and concerns among modelers as well as among experimentalists.

**Results:**

In this paper, we focus on homology-modeling and more specifically, we review how it is perceived by the structural biology community and what can be done to impress on the experimentalists that it can be a valuable resource to them. We review the common practices and provide a set of guidelines for building better models. For that purpose, we introduce the H-factor, a new indicator for assessing the quality of homology models, mimicking the R-factor in X-ray crystallography. The methods for computing the H-factor is fully described and validated on a series of test cases.

**Conclusions:**

We have developed a web service for computing the H-factor for models of a protein structure. This service is freely accessible at http://koehllab.genomecenter.ucdavis.edu/toolkit/h-factor.

## Background

### Introduction

Since 1958, when Kendrew et al reported the first atomic-level resolution of a protein structure (myoglobin), the structural biology field dramatically expanded with the development of new tools and methods to gain access into atomic details of a protein or a nucleic acid [1]. This opened a completely new world of knowledge and understanding to the scientific community, as the analysis of protein structures provides fundamental insight into most biochemical functions and consequently into the cause and treatment of diseases. Structural biology is now recognized as a fundamental step in our quest to understanding life at the molecular level.

Finding the structures of all proteins is currently a bottleneck for genomics studies. In this matter, the Protein Structure Initiative (PSI) aims at the determination of the three-dimensional (3D) structure of approximately 100,000 structures in 10 years. However, the protein sequence databank (UniProt/TrEMBL) is growing at a much faster rate, with more than 10 millions sequences available to date (March 2010). At the same time point, the Protein Data Bank (PDB) includes 64,100 structures, out of which only approximately 4300 are "unique" at chain level (i.e. once we remove "redundant" proteins whose sequences have more than 95% sequence identity with another protein in the PDB). It should be noted that these structures only represent a biased sample of the protein universe. For example, the PDB includes only 220 unique membrane proteins which is very little since membrane proteins constitute around 20-30% of most proteomes [2]. Noteworthy, the human genome has ~21,000 protein-encoding genes for a proteome of ~1,000,000 proteins when combining the complexity induced by alternative slicing events [3]. In addition, due to experimental limitations, the vast majority of the solved structures are below the 50 KDa threshold excluding numerous larger proteins. Large proteins however represent a significant fraction of the proteins present in an organism; for instance, proteins found in yeast *Saccharomyces cerevisiae *have five hundred amino acid residues on average and their lengths can reach two thousand eight hundred residues [4]. The structure of these large proteins, as well as of even larger assembly can be solved by electron microscopy at a somewhat low-resolution. While this field is expanding very fast and a growing number of structures solved at atomic-level resolution have been reported [5,6], its impact with respect to the size of the protein sequence databank remains limited. Many more protein structures have been solved by either X-ray crystallography or nuclear magnetic resonance (NMR). It remains however that most proteins are out-of-reach because of technical difficulties. There is clearly a huge gap between the world of known structures and the universe of known protein sequences. Structural genomic projects are unable to keep up with newly discovered genes.

One way to work around this problem is to use computational methods to predict proteins structures. *In silico *protein structure prediction techniques can be divided into two categories: the *ab-initio *folding methods and homology modeling. In this paper we focus on the latter. We note that both approaches have been shown to yield astounding results, as shown in the successive CASP contests [7]. However, they do require caution: while predicting the structure of a protein is an intellectual challenge that requires solving many practical issues, it is often considered as an art in essence.

The growth rate of structures deposited in the PDB is slowing down since 2004, along with the number of new superfamilies or folds discovered [8,9]. One possible explanation is that many proteins still evade the structural biology pipelines at this time because of the technical difficulties described above. In 1992 Chothia hypothesized that the number of protein folds in nature is probably finite and around 1,000 [9,10]. The latest analysis of the PDB and of the structural classification of proteins (SCOP) showed that we have not yet reached a plateau (currently estimated to be around 1,500) [8]. The current rate at which proteins are added in the PDB is far too slow to match with the number of new protein sequences discovered every year. The situation is however not so negative. There is a definite hope that the current content of the PDB will allow us to predict reliably the correct scaffold of more than 70% of the whole proteome using *in silico *methods [11]. This is the rationale for using homology modeling to complement experimental techniques.

**Figure 1:**
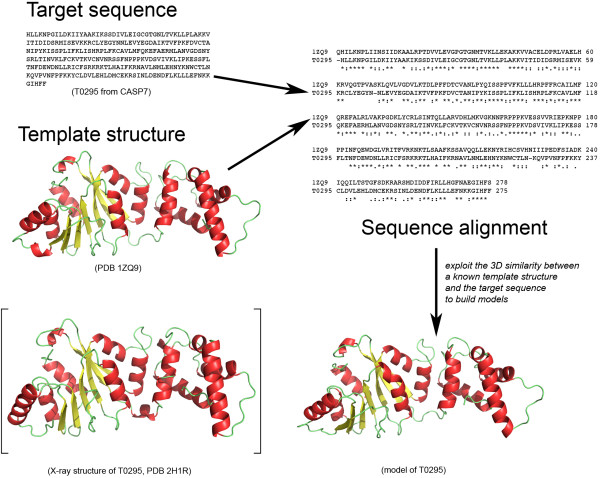
**A schematic flow chart of the homology modeling method**. Example of the T0295 target (Pf-KsgA from *Plasmodium falciparum) *from CASP7. The sequence alignment between the sequences of T0295 and the template protein in PDB file 1ZQ9 was generated using ClustalW V.2.0.10 [87] and the model was built using MODELLER 9v5 [36]. The X-ray structure of T0295 (PDB 2H1R) is displayed within brackets for visual comparison with one of the T0295 models.

Homology modeling predicts the structure of a protein by inference from a homologous protein whose structure is known (see Figure 1 for a schematic illustration of the technique). Its success rests on (i) the existence of a homologue with known structure, (ii) our ability to detect this homologue, and (iii) the quality of the model building process once the homologue is detected. Steps (i) and (ii) have greatly benefited from the different genomics projects: with the additional structures generated by the structural genomics projects, sampling of the protein structure space is becoming finer, improving the chance that a structural homologue exists for any given protein sequence. With the parallel increase in the number of sequences available in genomic databases and the development of meta-servers to analyze and query sequence databases, there has been significant improvement in the detection of homology [12]. In the recent CASP experiments, targets with sequence identity of 6% to their templates were included in the Homology Modeling category [13-15]. There is hope that in the near future homology modeling will reach its ultimate goal: the generation of model protein structures as accurate as those determined by high-resolution experimental studies.

Biologists unanimously consider X-ray crystallography as the prime source of structural information on proteins and the "gold standard" in term of accuracy: they base their confidence on its long list of published successes. The vast majority of structures deposited in the PDB were determined by X-ray crystallography and 14 Nobel prizes in Chemistry and Medicine have been awarded to crystallographers [16] (For recent reviews, see Kleywegt and Jones, 2002 [17]; Wlodawer et al, 2007 [16]; Brown and Ramaswamy, 2007 [18]; Ilari and Savino, 2008 [19]). Homology modeling on its own however is not devoid of successes. The very first published homology model in 1969 was the small protein α-lactalbumin, which was modeled on the basis of the structure of hen egg white lysozyme as a template [20] with the two proteins sharing 39% sequence identity. When the structure of α-lactalbumin was later solved by X-ray crystallography [21], the model turned out to be essentially correct [22]. Since then, homology modeling has continuously extended its field of applications, including designing mutants to test hypotheses about protein functions, identification of active sites, drug design, protein-protein docking, facilitating molecular replacement in X-ray structure determination, refining models based on NMR constraints (for recent reviews of homology modeling applications, see [23-25]). Despite these successes, homology modeling is not yet a well-established alternative or complement to experimental structural biology. It remains the focus of many criticisms often coming from the structural biologists themselves as they often consider a protein model to be unreliable, not being based on experimental data. The question arises as to what needs to be done to give homology modeling its credentials.

In this paper, we focus on homology modeling and more specifically, we review how the structural biology community perceives it and what can be done to impress on the experimentalists that it can be a valuable resource to them. It is organized as follows. The next section reviews the differences and similarities between homology modeling and high-resolution experimental structural biology. In particular, we illustrate steps in the homology modeling procedure that are putative source of errors. Our goal is to provide a useful step-by step handbook for non-specialists in order to help building better model.

The following section introduces the H-factor, a new indicator for assessing the quality of homology models. The H-factor is designed to check how well a family of homology models reflects the data that were used to generate those models, in the spirit of the R-factors in X-ray crystallography. The results section that follows validates the H-factor on a series of test cases. We conclude the paper with a discussion on what remains to be done to make homology modeling a prime technique for the biologists.

### Homology Modeling versus Experimental Structural Biology

This section reviews briefly the quality of protein structure models obtained either using high-resolution experimental techniques or homology modeling. Our hope is to identify common good practices as well as safeguards from which we can derive a validation tool for the latter. We start with the concept of a structural model and its meaning in the two communities of experimental and computational structural biologists. We then highlight the pros and cons of X-ray crystallography and NMR spectroscopy. An overview of the different steps involved in homology modeling follows, with emphasis on sources of errors and how they can be checked. Ultimately our goal is not to rank these methods but rather we hope to show that they all provide valuable and often complementary information, as long as the proper safeguards are applied.

#### What is a model?

The meaning of the word "model" is ambiguous in the structural biology community. A model for a protein structure can be obtained either by X-ray crystallography, by NMR spectroscopy, by electron microscopy, by computational methods or by combinations of all or some of these techniques. With experimental techniques, the atomic coordinates are refined against experimental structural restraints and constraints. Eventually the final model is called "structure" when the refinement statistics converge toward acceptable canonical values. Note that often this final model is subjected to refinement using simulation techniques such as constrained molecular mechanics or molecular dynamics simulations. Even though these simulations are constrained with the experimental data, the subsequent "structure" cannot be considered to be fully independent of modeling. On the other hand, an *in silico *model is generated without or with very limited experimentally constraints: it depends obviously on the hypotheses included in the modeling process, on the force-field used in the simulations as well as on the quality of the scientific computing tools that were used during the modeling steps. While the quality of an "experimental" model can be assessed directly against the experimental data, the quality of an "*in silico*" model is more subjective and ultimately defined through the usefulness of the model: this is most probably the source of the mistrust towards modeling in general.

#### X-ray crystallography: source of errors and quality metrics

A number of factors contribute to the quality of an X-ray structure. The first factor relates to the intrinsic crystal properties and its diffraction capabilities, which is mostly evaluated in term of resolution. The quality metrics used in X-ray crystallography fall into three categories: 1) to measure the quality of the raw data, 2) to measure the agreement of the refined structure against the data and 3) to validate only the model for ideal stereochemistry, rotamers and bad clashes implemented in What Check or Molprobity for instance. The first category lies upstream of the structure building process as the measures it includes evaluate the quality of the experimental diffraction data. The R_sym _indicator for example measures the average spread of individual measurements in respect to their symmetry equivalent measurements. A good dataset will have an R_sym _smaller than five percent. In addition, the quality of a dataset is also assessed by its signal-to-noise ratio <*I*/σ(*I*)> and its data-collection completion for a given space group. Unfortunately, only a few crystals diffract to atomic resolution (under 1 Å) with ideal quality metrics. Most of the crystal-based structures have therefore been solved with good to average raw data quality. Although building a structure can be semi-automatic with automated tools available for chain tracing, side chain-building, ligand building and water detection, it is still refined by experimentalists using their subjective interpretations of the data. It is common for example to find areas of poorly defined electron density map due to disordered regions. The experimentalist interprets these data to the best of her knowledge but this is unfortunately a common source of errors. The second set of quality metrics assesses the relative agreement of the structure in regard to the experimental data. This set includes the R-factor and the "free" R-factor (R-free). The R-free is analogous to the R-factor but uses a small subset of the data that have been flagged-out and not taken into account during any refinement process [26]. Its purpose is to monitor the progress of refinement and to check that the R factor is not being artificially reduced by the introduction of too many parameters. As such, it provides an unbiased indicator of the errors in the structure and prevents any over-refinement and over-interpretation of the data. Both factors along with R_sym _and R_merge _can be seen as indicators of the errors inherent in the refined model and in the experimental data.

The quality of protein crystal structures has been reviewed several times over the last fifteen years and it is striking to notice that despite a constant increase of the technology and validation tools, it has not improved overall. The quality spectrum of X-ray structures remains broad. The increase of automation through structural genomic pipelines did not help raising the bar in that matter as human intuition and reasoning are taken out of the process [17,18]. Interestingly, X-ray structures published in high-impact general science journals are usually the worse offenders in term of quality and errors. This is explained by the experimental difficulties associated with solving novel high profile structures and the rush to publish in a competitive environment [18].

X-ray crystallography is not immune to errors and mistakes, both honest and dishonest. Unfortunately, over the years we have seen gross mistakes in various structures leading to the retraction of several high-impact papers in leading journals because of a lack of quality control during the structure building pipeline [27,28]. A recent review by G. J. Kleywegt highlights the need and the proper use of validation methods in structural biology in general and in X-ray crystallography in particular. The author also emphasizes the use of validation methods early on in the project pipeline in order to minimize the number of erroneous high-profile structures that can hinder the progress of science for years to come [29]. In light of a growing number of structures falsification and to prevent both dishonest and honest mistakes in structures determination, the curators of the PDB have implemented over the years new sets of validation procedures for the deposition process [30].

#### NMR spectroscopy: source of errors and quality metrics

Although structure determination by NMR spectroscopy methods is very different from X-ray crystallography, it shares similarities with the latter in terms of sources of errors. Instead of using an X-ray beam diffracting around electrons in a crystal, NMR spectroscopy is performed in solution and uses the magnetic properties of the nuclei with odd spins (mostly ^1^H, ^13^C and ^15^N). As the molecule of interest is placed in a strong magnetic field, each of these nuclei is characterized by a unique resonance frequency, i.e. the frequency at which it will absorb energy. This frequency depends on the local magnetic field that combines the external field and the local environment: it is referred to as the chemical shifts. NMR experiments are designed to monitor the behavior of these nuclei as the system is perturbed from equilibrium and each experiment usually isolates one property, such as through-bond connectivity's (COSY and TOCSY experiments) or spatial proximity that allows for energy transfer (NOESY experiments). In the specific case of proteins, the number of nuclei involved can be large, leading to crowding of the spectra: this is usually overcome by using multidimensional experiments (mostly 2 D, but also 3 D and 4D). The typical protocol for protein structure determination by NMR proceeds as follows. Firstly, the chemical shifts observed on multidimensional spectra are assigned to their specific atoms (nuclei) in the protein (the assignment process). Second, through-the-bond and through-space coupling effects (i.e. J-coupling and Nuclear Overhauser effects, respectively) observed on these spectra are quantified and concerted into angles and distance restraints. Most of these restraints correspond to ranges of possible values instead of a precise constraint. Thirdly, a molecular modeling technique is used to generate a set of models for the protein structure that satisfy these experimental restraints as well as standard stereochemistry. For a more detailed presentation of the application of NMR to protein structure determination, we refer the reader to [31-34]. Analogously to X-ray methods, the quality of NMR measurements affects the quality of the structures. The precision of a set of models for a protein structure determined by NMR is evaluated as the root-mean-square (RMS) difference between each model and a "mean" structure, defined geometrically as the mean of all the models (note that the stereochemistry of this mean model is usually not correct). The quality of each model is evaluated by the number of violations observed in the model compared to the experimental restraints. A high-quality NMR structure refers to a set of high quality models with no violations that are tightly bundled around their mean, i.e. with a small RMS. Note that in addition to these NMR specific quality measures, Garrett and Clore introduced a R-factor and a free R-factor for the refinement of NMR structures based on residual dipolar coupling, a long range NMR measure obtained on proteins that have been partially oriented in dilute liquid crystals. In similarity with X-ray crystallography, the quality spectrum of NMR structures is broad, with errors and mistakes reported that are inherent to a human based determination process [35].

#### Homology modeling: source of errors and quality metrics

The general strategy developed for homology-modeling proceeds through a canonical seven-steps procedure (Figure 1): (1) Identify the template proteins that share structural similarity to the target; (2) Align the target sequence with the templates sequences; (3) Build a single framework of spatially aligned template structures and assimilate the target protein backbone with this framework; (4) Build the missing backbone elements (loops) not represented in the template framework; (5) Build the target side chains; (6) Refine the model in order to minimize unrealistic contacts and strains; and (7) Evaluate the final refined model for physical tenability. To date numerous homology-modeling programs such as MODELLER [36], SegMod/ENCAD [37], Swiss-model [38], 3D-Jigsaw [39], BUILDER [40] and Nest [41] have been developed and many of them have been embedded into homology-modeling servers to ease the burden of generating models. Online portals such as the Protein Structure Initiative (PSI) model portal or the Swiss-Model Repository bring to the community a large database of models [42]. The PSI model portal currently provides 8.2 millions comparative protein models for 3.1 million distinct UniProt entries. Every model comes with relevant validation data. However those models are automatically generated without any human interaction that might render them inaccurate without any extra validation steps. In the following, we overview each step of the homology modeling process and highlight potential sources of errors.

#### *Steps 1 and 2: template selection and sequence alignment

The selection of template(s) is undoubtedly a critical step in modeling. It was long assumed that two proteins whose sequences share at least 40% identity have similar structures. If such a template exists, it is easily detected by any sequence alignment techniques. Homology modeling under such conditions is then expected to generate models whose accuracy is close to that of an experimental structure. We know however that this is not always true. Roessler et al. recently reported the discovery of two native Cro proteins sharing 40% sequence identity but with different folds [43]. Moreover, Alexander et al. were able to design two proteins with 88% sequence identity but having different structure and function [44]. Reversely, it is not uncommon for proteins, especially enzymes carrying the same function across the tree of life to share a somewhat low sequence identity and at the same time being structurally similar, with a Cα r.ms.d. ~1.5Å [45]. All these observations indicate that the selection of template is far from being a trivial task and extreme caution should be applied.

The situation is even more difficult if there is no significant sequence similarity between the sequence of the target protein and any of the known protein structures. This is one of the current challenges of the post-genomic era that is tackled by fold-recognition methods, namely to identify a suitable template for homology modeling [46-48]. Unlike sequence-only comparison, fold-recognition techniques take advantage of the information made available by 3 D structures. Despite a steady development over the years, as illustrated through the successive CASP experiments, fold recognition techniques still have a number of limitations. They are however the key to extend the domain of application of homology modeling methods.

**Figure 2:**
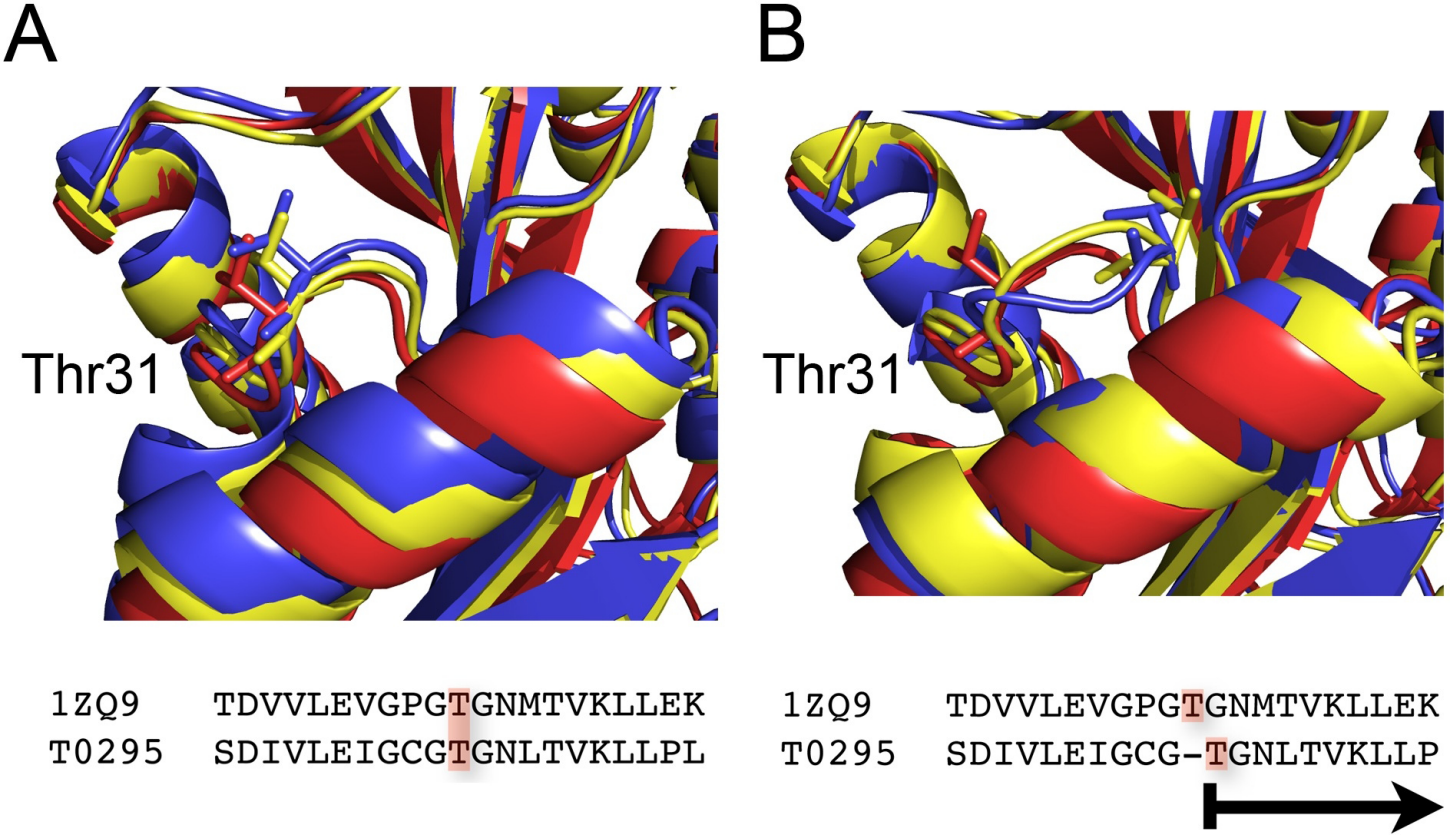
**Impact of a misalignment in homology modeling**. The target protein T0295 from CASP7 is modeled using the crystal structure of human dimethyladenosine transferase (PDB 1ZQ9) as a template. In panel (A), we show the structural alignment of the template 1ZQ9 with the best (yellow) and worst (blue) models generated by MODELLER 9v5 for T0295. The structural diversity between these models is low; Thr31 for example superposes very well in the three structures. In panel (B), we show the effect of an error in the sequence alignment that serves as input to the modelling process. We shifted the alignment by one residue at the level of Thr31, and generated new models for T0295. The superposition of the template 1ZQ9 with both the best and the worst models shows locally a structural heterogeneity in the loop that contains Thr31. Thr31 is being shift by 3.4 Å due to one single error in the sequence alignment.

The accuracy of the sequence alignment is another critical step in the homology modeling process [49]. It is important to keep in mind that a shift of one residue in the alignment introduces a distortion of 3.8 Å in the model for the backbone of the target protein (Figure 2A &2B). Therefore, it is of paramount importance to precisely verify and locally refine the sequence alignment used to build a model. For that matter, the areas of sequence comparison and alignment optimization have grown in parallel to the comparative modeling field [50]. There are many ways to align protein sequences and numerous methods to score the accuracy of the resulting alignments. Homology modeling usually relies on one of following three techniques: (a) standard pairwise sequence alignment using dynamic programming, (b) multiple sequence alignment when the target and template sequences belong to a large family for which many sequences and structures are known, and (c) direct alignment of the sequence on the structure of the template using a threading technique. None of these techniques is the panacea, especially when the sequence identity between the target and template sequences is low. Errors come for example from the fact that the optimal alignment between the sequences of two proteins may not match the alignment between their structures: this is caused by the sequence alignment technique that optimizes a score based on a substitution matrix and gap penalties while structural alignment techniques optimize geometric matching. There is no reason that these two different metrics are equivalent. Errors are often frequent in the loop regions between secondary structures, as well as in regions where the sequence similarity is low. Recent techniques that attempt to reduce the error rate in the sequence alignment rely on the inclusion of as many sources of information as possible, such as amino-acid variation profiles, secondary structure knowledge, structural alignment data of known remote homologues, knowledge of "anchor regions" (active-site catalytic residues or conserved motifs based on biological data). Many programs also attempt to optimize the raw sequence alignment derived from one of the techniques described above. Most of these programs have been embedded into web-servers for ease of uses, such as MUSCLE [51] and Dialign-TX [52].

#### * Step 4: Loop building

The loop-building step is another key component in homology modeling. Loops participate in many biological events and functional aspects such as enzyme active sites, ligand-receptor interactions, and antigen-antibody recognition among others. However, due to the flexible nature of loops, it is often difficult to predict their conformation. There are two main approaches to tackle the problem of loop modeling: methods that use databases of loop conformations or *ab initio *methods. In the database approach, a library of protein fragments whose size corresponds to the size of the loop to be modelled is scanned for fragments whose end-to-end distance matches the corresponding distance in the framework. The library is derived from the known protein structures in the PDB. This method has proved to be accurate when the loop is relatively short. Fidelis et al. have shown that loops of a maximum of seven residues can be modelled with confidence based on known structures [53]. When the database method is combined with a restrained energy minimization, it extends the confidence of loop building up to nine residues [54]. Beyond the nine residues threshold, *ab initio *methods have to step in mostly because for these longer loops, the fragment library provides a poor sampling of the conformational space accessible. The *ab initio *loop prediction approach relies on a conformational search guided by a scoring function. The accuracy of *ab initio *loops modeling remains currently low, especially when dealing with very long loops [55].

#### * Step 5: The side-chains positioning problem

**Figure 3:**
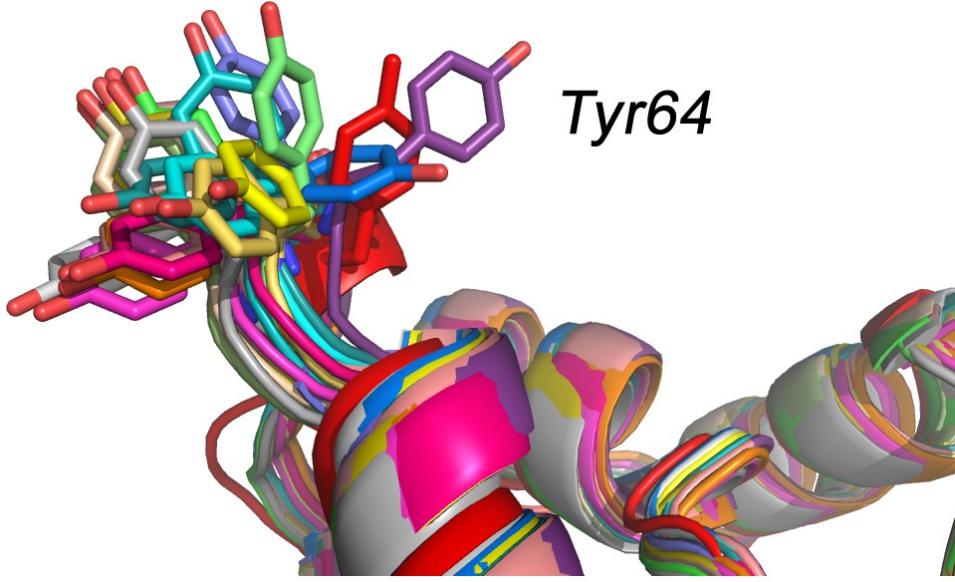
**The side-chains positioning problem**. We compare 10 models constructed for target T0295 with the native structure of T0295, available in the PDB as file 2H1R (shown in red). We show only the backbones of each model and the native structure, except for the sidechain of Tyr64. The diversity observed for the conformation of this sidechain emphasizes the difficulty to model sidechains accurately.

The prediction of side-chain conformations for a given backbone architecture remains a challenge. It is however the key to generating models at atomic resolution. For instance, critical side-chains forming an active site must be accurately positioned in order to support any putative catalytic mechanism. Nearly all the side-chain positioning methods are based on rotamer libraries with discrete side-chains conformations. Rotamer libraries contain a list of all the preferred conformations of the side-chains of all twenty amino acids, along with their corresponding dihedral angles [56,57]. Some of these rotamer libraries are further refined to account for the local geometry of the backbone [58,59]. Sidechain prediction techniques select the best rotamer for each residue of the protein under study from one of these libraries, based on a score that includes both geometric and energetic constraints. This leads to a large combinatorial problem: see Figure 3 for an example of the size of the conformational space accessible to a sidechain in a protein, even when we discretize this space by using rotamers. The combinatorial problem is solved by heuristic techniques such as mean field theory, derivatives of the dead-end elimination theorem or Monte Carlo techniques [60]. The success rates of the most successful techniques range from 78% to 89% for the χ^1 ^and χ^2 ^angles of residues in the core of the protein (i.e. residues whose solvent accessibility is less than 30%). It is important to note that these results usually relate to mock experiments in which the exact conformation of the backbone is known. It has been shown that the quality of sidechain prediction decreases as the deviation between the backbone used for modeling and the actual conformation of the backbone increases [58,61]. These results emphasize the need of a good framework (and implicitly of a good sequence alignment between the target and template protein) for homology modeling. It is worth mentioning however that a 1Å accuracy over the main-chain atom position can be related to X-ray structures with 2.5 Å resolution and with a R-factor around 25%, which is fairly common [62]. In addition, multiple side chain orientations are routinely observed in crystal structures and crystal-packing forces can alter their positions as well. The side chain prediction methods are improving steadily, as reported by the CASP experiments [63].

#### * Step 6: Refinement of the final model

In a review written in 1999 on the CASP3 experiment, Koehl and Levitt noted that most models submitted in the homology modeling category were not refined, as previous CASP meetings had shown that refinement did not improve the models [64]. Sadly for the computational biologists the situation has not improved and it remains difficult to generate a model closer to the native structure than the template used to build it [13]. Energy refinement, originally introduced by Levitt and Lifson forms the basis of current methodologies for protein structure refinement against experimental data [65]. Without experimental restraints, refinement by energy minimization generally moves the protein structure away from its X-ray structure. Some recent studies have shown that this negative trend can be reversed through the inclusion of evolutionary derived distance constraints [66] through the combination of sophisticated sampling techniques based on replica exchange molecular dynamics and statistical potentials [67], through the addition of a carefully designed, differentiable smooth statistical potential [68], or by careful consideration of the solvent effects [69]. While these studies are definitely source for hope, much work remains to be done as far as refinement is concerned.

#### A general framework for model assessment: R-factors and equivalent

The wide availability of homology modeling software packages, as well as the development of web interfaces that automate the use of these packages has resulted in better access to and a broader usage of homology modeling. While this is definitely commendable and homology modeling should be even more advertised, there are risks that this will lead to errors because of the difficulties in evaluating the correctness of the models these techniques generate. This is primarily due to the lack of cross validation indicators such as the R-factor and R-free in X-ray crystallography [70]. In addition to the stereochemistry assessment of the structure and the good correlation between the R-factor and the R-free values, the quality of an X-ray structure can be evaluated based on the thermal motion value of atoms described by the Debye-Waller factor or B-factor. The B-factor allows for the identification of zones of large mobility or error like disordered loops. When multiple monomers populate an asymmetric unit of a crystal, the crystallographer will choose to focus on the analysis of the monomer with the lowest average B-factor since the likelihood of errors is lower. Unfortunately, such criteria do not apply to models thus rendering the identification of zones of uncertainties a non-trivial task.

With respect to homology modeling, the main step is to thoroughly validate the model and always provide all the relevant details about the protocol used. This will give the user all the necessary data to judge the quality of the model. The quality assessment of models has been the focus of numerous studies and various algorithms have been reported over the years. In this matter, tremendous efforts are being made to produce the best triage procedures or scoring functions among models, as seen in the latest CASP meetings [71]. These scoring functions are based on statistical potentials [72], local side-chain and backbone interactions [73], residue environments [74], packing estimates [75], solvation energy [76], hydrogen bonding, and geometric properties [77]. In addition, it is essential that the quality of stereochemistry be kept high. The stereochemistry can be assessed by commonly used programs such as Procheck or WhatIf [78,79].

Ultimately, the validation of models comes from experiments such as site-directed mutagenesis, circular dichroism, cross-linking, mass spectrometry, fluorescence-based thermal shift, light scattering, molecular FRET or electron microscopy. Such experimental data can be translated into constraints/restraints and introduced in the modeling protocols thus improving the accuracy of models. One can also identify fast and cheap experimental procedures that can help testing homology models. The easiest way is to crosscheck models with experimental structures. For instance with enzymes, it is possible to verify the location of important catalytic residues in the active site by comparison with homologous family members. Most importantly however, a model needs to be checked manually in the same way a NMR or an X-ray structure is processed.

Despite all these methods, the homology modeling community still lacks a simple indicator which gives an unambiguous feedback on how the final model, or family of models, reflects the data that were used in the modeling process, similar to the couple R-factor/R-free for X-ray crystallography. The next section introduces such an indicator, namely the H-factor.

## Methods

### Computing the H-factor

**Figure 4:**
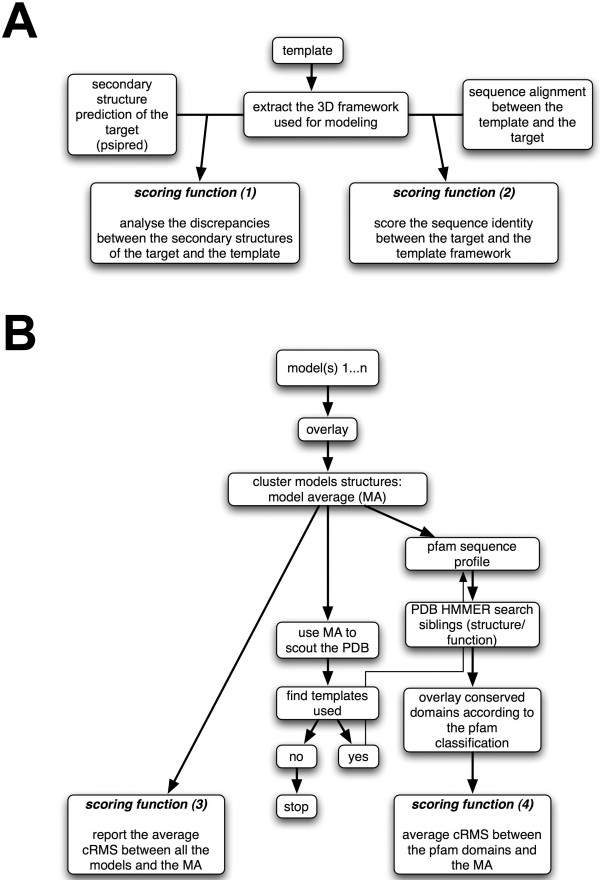
**Flowchart for computing the H-factor**. **A**. The scoring functions ***(1) ***and ***(2) ***are sequence-based: score ***(1) ***compares the secondary structure prediction for the target sequence with the actual secondary structure assignment of the template protein, while score ***(2) ***evaluates the sequence similarity between the target and template sequence. **B**. The scoring functions ***(3) ***and ***(4) ***evaluate the structural models: score ***(3) ***quantifies the structural diversity among the models, while score ***(4) ***identifies the pfam domain in the target protein, collects the structures of these domains from the models to be tested, and compares these structures with those observed for the same domains in the PDB.

In this study, we introduce a novel indicator as an attempt to quantify how well a homology model correlates with the input data and its biological relevance. This indicator, the H-factor is designed to mimic the R-Factor in X-ray crystallography. It is rooted in the basics of homology modeling and it uses all data that were included in the model building process to assess its correctness, in addition to checking for good stereochemistry. More specifically, the H-factor combines information on ***(1) ***the template structure(s) (based on the corresponding PDB files); ***(2) ***the sequence alignment between the template(s) and the target sequences; ***(3) ***the structural heterogeneity of the models built; and ***(4) ***the structural neighborhood within protein families. Note that at this stage, the computation of the H-factor is based on a single framework. We plan to derive an extended H-factor that will account for multiple templates. Each of these four categories is assigned a scoring function returning a value between 0 (best) and 10 (worst). The H-factor is simply the sum of these scoring functions divided by 40. For ease of use, the H-factor is converted into a percentage with a value between 0% (ideally the perfect score) and 100% (the worst score). In par with the R-factor in X-ray crystallography, a low H-factor value indicates a trustworthy set of models, while a large H-factor would raise a flag on the correctness of the models. The H-factor focuses on the Cα-backbone as a correct tracing is a prerequisite for a valid model. The workflow of the H-factor calculation is detailed on Figure 4A and 4B.

The scoring function ***(1) ***analyses the discrepancies between the secondary structure prediction for the target obtained with the program psipred [80,81] and the actual secondary structures of the template framework computed with the program stride [82]. The corresponding score takes into account the confidence factors reported by psipred:

$$\begin{array}{l} {\text{score}}_{1}=a\sum_{i=1}^{N}\frac{f(i)}{N}+b\\ f(i)=\{\begin{array}{ll} 0 & \text{for}\;p=s\\ \frac{c(i)+1}{10} & \text{for}\;\text{p}\neq s\\ & \text{for gaps in}\\ 1 & \text{the}\;\text{sequence}\\ & \text{alignment} \end{array} \end{array}$$

where the sum is computed over all positions in the sequence alignment between the target and template, *N *is the length of the sequence alignment, *p *is the secondary structure prediction of the target at position *i *(values for p are 'H' for helix, 'S' for strands, and 'C' otherwise), *c(i) *is the confidence factor reported by psipred (integer value, from 1 to 10) for the secondary structure prediction at position *i *and *s *is the secondary structure type observed at position *i *in the template structure reported by stride. The offset coefficients *a *and *b *are set to 1.3 and 0.9, respectively, to ensure that score ***(1) ***has values between 0 and 10.

The function ***(2) ***scores the identity between the sequence of the target and the sequence of the template framework (Cf. Figure 4A).

$$\begin{array}{l} {\text{score}}_{2}=10\left(1-\frac{\sum_{i=1}^{N}g(i)}{N}\right)\\ g(i)=\{\begin{array}{ll} 0 & \text{for non identity}\\ 1 & \text{for identity} \end{array} \end{array}$$

where *N *is the length of the sequence alignment.

The search for a template structure for a given target protein sometimes finds multiple candidates. In addition, considering multiple options for this alignment sometimes alleviates ambiguities in the alignment between the sequence of one of these candidates and the sequence of the target. Both situations lead to multiple structural models being generated for the target protein: score ***(3) ***is designed to measure the heterogeneity of this set of models. It uses as input all the models M_i_, as well as the corresponding average model, MA, whose atomic coordinates are the averages of the corresponding coordinates in the models. The average model MA is computed from the structural alignment of all the models, which can be computed for example with the *cluster() *function in MODELLER [36] or with the program maxcluster [36,83]. The function ***(3) ***then reports the average cRMS between each model and the average model, where the cRMS is computed over the Cα atoms only. The average cRMS is then transformed linearly such that the final score is between 0 and 10:

$${\text{score}}_{3}=a\left(\frac{\sum_{\text{i}=1}^{n}\text{cRMS}({\text{M}}_{\text{i}}\text{,MA})}{n}\right)+b$$

*n *is the number of models. The offset coefficients a and b are chosen such that average RMS values of 0.1 and 7 Å correspond to scores of 1 and 10, respectively; the corresponding values are *a *= 1.3 and *b *= 0.87.

The search for a template structure sometimes finds partial matches. In many cases, this is related to the fact that the target protein contains multiple functional domains that may not always be associated in other proteins. While the modeling is performed based on the longest possible template, it is expected that each of these domains have been correctly modelled. Score ***(4) ***is designed to quantify this assertion. It first identifies the various domains in the sequence, using HMMER [84,85] and the pfam profiles database as a reference [86], The average model MA is then broken down into fragments corresponding to the domains that have been identified. Each fragment is compared to the structure of the same domain found in proteins whose structure has been deposited in the PDB. A maximum of 5 fragments is considered (the top 5 HMMER search results). To minimize the numbers of false positive we set an E-value cut-off of 1.0e-10 for hmmsearch. The score ***(4) ***is then the average cRMS distance between the fragments and their counterparts in the PDB:

$${\text{score}}_{4}=a\left(\frac{\sum_{\text{d}=1}^{m}\sum_{\text{i}=1}^{n}\text{cRMS}({\text{MA}}_{\text{d}}{\text{,D}}_{\text{d,i}})}{mn}\right)+b$$

*m *is the number of functional domains identified in the target sequence, MA*_d _*is the structural fragment extracted from the average structure MA corresponding to the domain *d*, *n *is the number of domains homologous to domain *d *found in PDB structures, and D_d,i _is the i-th possible structure of the domain homologous to *d*. The offset coefficients a and b have been chosen such that average RMS values of 0.1 and 7 Å correspond to scores of 1 and 10, respectively; the corresponding values are *a *= 1.3 and *b *= 0.87. This usually enforces that score ***(4) ***is between 0 and 10. Note that if this procedure does not find an equivalent domain for a fragment, the fragment is ignored; if no domains are found for all fragments, score *(**4**) *is ignored.

The H-factor computation is accessible online at http://koehllab.genomecenter.ucdavis.edu/toolkit/h-factor with a simplified operating manual (Cf. additional files). The source code is available upon request.

### Testing of the H-factor on CASP targets

Target proteins that have been submitted to CASP in the homology modeling category are perfect tests for the H-factor measure presented here. The web site for the Protein Structure Prediction Centre that hosts all information about the CASP experiments http://predictioncenter.gc.ucdavis.edu also includes a database of all the models that have been submitted in the successive CASP with the corresponding experimental structures. This database of CASP models contains models that can be ranked from being very accurate to being completely wrong. It is therefore ideally suited to benchmark modeling techniques and to develop and test validation methods. We have chosen three targets from the recent CASP7 experiments: T0287, T0375 and T0295 that have been identified as homology modelling targets by the CASP organizers (see table 1 for details). The first target (T0295) was considered "easy", while the second and third target (T0287 and T0375) were more difficult cases for homology modeling. Fold recognition techniques initially identified one template for T0287 and T0295, and six potential template structures for T0375. The top templates were then selected according to the CASP7 analyses of every possible template for each target. We aligned the sequence of the target with its template(s) using ClustalW [87], with the Gonnet250 matrix to define the substitution score and default settings for gap penalties (Cf. additional files 1, 2). For the first two targets, this corresponds to a simple pairwise alignment, while for T0375, ClustalW provides a multiple sequence alignment over the target sequence and the six possible template sequences. We used MODELLER 9v5 [36], with the "automodel" settings to generate 20 models for each of the three targets. As the H-factor focuses only on the Cα-backbone, we did not attempt to improve the prediction of the sidechains. In addition, we did not perform any energy minimization of the final model and it still remains unclear if energy minimization improves models generated by homology modeling [88].

**Table 1:** Test set based on CASP targets.

Test case (CASP ID)	*Template(s) (resolution (Å))*	*%sequence identity between template and target*	*Coverage*	*Type of sequence alignment*	*Loop segments of 3 or more residues*
T0295	1ZQ9 (1.90)	46	1-275 (100%)	pairwise	80-86; 163-166; 179- 182; 225-228; 271-275
T0375	2DCN (2.25); 1RKD (1.84); 1V1A (2.10); 1VM7 (2.15); 2AFB (2.05); 2FV7 (2.10)	17	1-296 (100%)	multiple	33-37; 75-78; 100-103
T0287	1V55 (1.90)	16	1-199 (100%)	pairwise	33-37; 75-78; 100-103; 166-170; 197-199

## Results and discussion

### The H-factor: detailed analysis on three CASP targets

The CASP7 target T0295, a dimethyl adenosine transferase from *Plasmodium falciparum *is considered an "easy" modeling case: it has a very close homologue (sequence identity 46%) whose structure has been solved at high resolution (PDB code: 1ZQ9, 1.9 Å resolution) and in addition the template sequence covers the whole sequence of T0295. We generated 20 models for T0295 using standard homology modeling techniques (see methods). In this experiment, our goal was not to generate the ultimate, high-resolution model. Instead, we were interested to see if the H-factor we have introduced was able to measure the quality of the models we generated. The average CRMS (based on Cα only) between the T0295 models and the actual experimental X-ray structure for target T0295 (available in the PDB as 2H1R; resolution 1.89 Å) is 1.67 Å: this clearly indicates that the models are of good quality. The corresponding H-factor for these 20 models is 19%, i.e. a very good score (by definition, H-factors vary between 0% and 100%, with 0% being good and 100% being bad). The good quality of the T0295 models is highlighted by each scoring function included in the H-factor (Table 2). The secondary structure prediction for T0295 matches well with the actual secondary structure of its framework (1ZQ9), yielding a value of 1 for score ***(1)***. The sequence alignment between T0295 and 1ZQ9 is deemed good, with a value of 1.9 for score ***(2)***. The 20 models generated showed little structural dispersion with a corresponding value for score ***(3) ***of 1.3. T0295 contains one pfam domain, PF00398 that corresponds to the ribosomal RNA adenine dimethylase. In addition to being found in the structure 1ZQ9, this domain is also found in the proteins whose structures are available in the PDB files 1I4W, 1QAM, 1QYR, 1YUB and 2ERC. Score ***(4) ***compared the structure of this domain in the models generated for T0295 and the structures of the same domain found in all 5 proteins listed above. It detected fluctuation between these structures, leading to a score of 3.8. Note that the pfam domain PF00398 occurs in species covering all three kingdoms. In addition, the structures have been derived from data with wide range of resolution (from 2.0 Å to 3 Å for the X-ray structure; 1YUB is an NMR structure). It is therefore not surprising that score *(**4**) *is relatively higher than the other scores; it does remain however within a range that indicates a good match. Note that the overall H-factor value is 19%. In comparison, a R-factor of 20% is typically observed for fully refined X-ray structures around 2 Å of resolution, i.e. for a good X-ray structure.

**Table 2:** Comparing the H-factor with cRMS, DOPE and QMEAN scores to assess models generated for CASP7 targets.

CASP 7 target	Scoring function (a)				H-factor (%)	cRMS (Å) (b)	% ID (c)	DOPE (d)	QMEANnorm (e)
	*(1)*	*(2)*	*(3)*	*(4)*					
T0295	1.0	1.9	1.3	3.8	19	1.67	46	-33940	0.735
T0295* (f)	1.0	1.9	1.9	3.8	21	1.71	46	-24317	0.196
T0375	1.1	8.6	2.9	3.8	41	3.16	17	-28442	0.546
T0287	7.0	8.1	7.5	7.5	75	5.81	16	-18444	0.285

(a) The four scoring functions included in the H-factor measure the quality of the secondary structure prediction (score ***(1)***), the diversity of the sequence alignment (score ***(2)***), the structural diversity of the models generated (score ***(3)***), and the similarity of the predicted structures for the functional domains in the target, compared to the structures of the same domains found in the PDB (score ***(4)***) (see text for detail)

(b) Average cRMS (over Cα) between the 20 different models generated and the actual experimental structure for the target.

(c) Sequence identity between the target sequence and the template sequence

(d) DOPE scores from MODELLER [94]

(e) QMEAN normalized score [95]

(f) T0295* is the toy experiment in which the sequence alignment between the target T0295 and its framework has been deliberately modified (see text for details).

When we deliberately introduce a shift at position 31 in the alignment between the sequence of T0295 and the sequence of its template 1ZQ9 (see Figure 2), the corresponding models generated by MODELLER show structural diversity in the loop region near the shift (i.e. near Thr31). Score ***(3) ***captures this structural diversity within a set of models. It leads to the H-factor being raised from 19% to 21% (see table 2). However, score ***(2) ***could not detect a single position shift in the alignment. The H-factor is therefore capable of detecting backbone deviation due to modeling errors, the same way the R-factor does.

The CASP7 target T0375 is a more difficult modeling case. It is a human ketohexokinase and the rigid-body domain closure of sugar kinases is known to be large, adding complexity into the modeling process [89]. Although several sugar kinase structures have been solved, the search for templates for T0375 identified only six distinct remote templates. Moreover, all six templates are needed to obtain complete sequence coverage of T0375 within one single framework with MODELLER. In addition, the template sequences have low similarity with the target sequence. This is detected by the scoring function ***(2)***, which returns a value of 8.6 (out of 10) (table 2). Note that the score ***(2) ***is not a direct measurement of the quality of the sequence alignment. It is designed to quantify the difference between the two sets of sequences: if this difference is small, the model is expected to be good, while if the difference is large, the sequence alignment most probably belongs to the twilight zone and the models should then be considered with caution. The overall H-factor for the models generated for T0375 is 41%. This mid-range value indicates that caution should be used when interpreting or using these models. Indeed, the average cRMS between these models and the actual structure of T0387 (available in the PDB in the file 2HLZ) is 3 Å, i.e. reflecting a medium-resolution agreement.

The CASP7 target T0287 is the most difficult test case we have considered. In fact, it would not be considered a homology-modeling target by many, despite the fact that a (remote) structural homologue is available in the PDB. We did decide to include it in our study to test whether the H-factor was still providing useful information when applied on a difficult test case. T0287 corresponds to CaGS, a protein from *Helicobacter pilori *whose function is unknown. A database search over all sequences of proteins whose structure is known identifies a unique template, 1V55, with a low sequence identity (16%, see table 1). 1V55 is a Cytochrome C Oxidase and it is not clear that T0287 and 1V55 are homologues. We did build 20 models for T0287, using 1V55 as a template, and the out-of-the-box alignment between the sequences of T0287 and 1V55 generated by ClustalW. As mentioned above, we did not try to optimize the alignment or the modeling itself as our interest is to see if the H-factor is able to assess the quality of the models we generated. In this specific case, all four scores reported high values (7.0, 8.1, 7.5 and 7.5 for scores ***(1)***, ***(2)***, ***(3) ***and ***(4)***, respectively). The overall H-factor is 75%, a valued that should raise concerns about the quality of these models. As for target T0375, this is confirmed by the experiment: the average cRMS between these models and the actual structure for T0287 (PDB code 2G3V) is 5.8 Å, indicating that the models are poor approximations of the native structure.

### H-factor: Detecting bad models

From the three test cases T0295, T0375 and T0287, we conclude that the H-factor correlates well with the quality of the models tested. To further confirm that high H-factor values correspond to models that should be considered with caution, we downloaded sets of models for seventeen target proteins from CASP7. These targets were considered as difficult, with only remote templates available. The corresponding sets of models, available from the CASP web site have poor to bad quality. When compared to the actual experimental structures, these models have a very high cRMS indicated major errors in the backbone tracing. In all sixteen cases, the H-factors are found to be high, as shown in table 3.

**Table 3:** Comparison of the H-factor with cRMS, DOPE and QMEAN scores to assess models generated for CASP7 targets from the free-modeling category

CASP 7 target	H-Factor (%)	cRMS (Å) (a)	DOPE (b)	QMEANnorm (c)
T0356_D1	48	4.9	-8908	0.242
T0356_D2	48	4.9	-13013	0.209
T0316_D3	49	6.0	-5488	0.176
T0356_D3	49	4.9	-9274	0.285
T0316_D2	51	5.8	-2980	0.110
T0307_D1	62	5.5	-12623	0.385
T0307	63	6.0	-13816	0.407
T0309	65	5.5	-4613	0.180
T0314	65	5.4	-9248	0.372
T0296	67	5.6	-35782	0.260
T0299	67	5.9	-17649	0.334
T0306	68	4.8	-7884	0.287
T0316_D1	68	5.6	-16102	0.214
T0299_D2	69	6.0	-7742	0.302
T0316	69	3.8	-31249	0.172
T0299_D1	70	6.2	-7641	0.250

(a) cRMS (over Cα) between the average model and the actual experimental structure for the target.

(b) DOPE scores from MODELLER [94]

(c) QMEAN normalized score [95]

### H-factor: characterizing good models

The H-factor computes the quality of models for protein structure based on sequence information (score ***(1) ***and score ***(2)***), as well as based on structure information (score ***(3) ***and score ***(4)***). While the former is specific to homology modeling, the latter can be used to assess the quality of any sets of models. We tested score ***(3) ***and score ***(4) ***on a random set of NMR structures for which 10 or more models are available. In this validation case, the H-factor is the sum of functions ***(3) (4) ***divided by 20 instead of 40, and converted to percentage. Results are listed in Table 4. In every case, the H-factor is low and below 35% in average, as anticipated. This indicates that the algorithm recognized the experimental structures as valid.

**Table 4:** H-factor applied to NMR structures with 20 models or more.

PDB id	Scoring function (a)				H-factor (%)	cRMS (Å) (b)
	*(1)*	*(2)*	*(3)*	*(4)*		
1A5E	1.03	n/a	2.13	3.43	**28**	**1.95**
1A2I	1.24	n/a	1.48	3.8	**34**	**2.29**
1A24	0.74	n/a	2.27	4.5	**38**	**2.79**
1A57	1.38	n/a	2.33	4.67	**35**	**2.91**
1A5J	0.99	n/a	2.38	4.67	**35**	**2.91**
1A67	0.37	n/a	2.67	4.8	**37**	**3.03**

**(a) **See text for the description of the scoring functions ***(3) ***and ***(4)***; these two functions measure the structural dispersion of the models considered, as well as their similarities to homologue structures in the PDB; note that scores ***(1) ***and ***(2) ***usually present in the H-factor are meaningless for this test set.

**(b) **Average RMS between each model and the "mean" structure computed from all the models.

**Figure 5:**
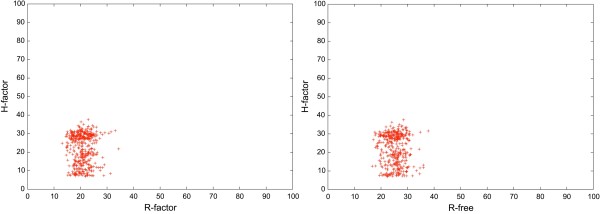
**Comparison between the H-Factor and the experimental R-factor and R-free**. A set of 445 randomly chosen X-ray crystallography structures of 6 or more identical chains have been scored with the H-factor and results are compared to both the experimental R-Factors and R-Frees. R-values are scaled in the interval [0,100%] to allow for direct comparison. Note that we computed a H-factor only from functions ***(3) ***and ***(4) ***since scores ***(1) ***and ***(2) ***that are usually in the calculation of the H-factor are meaningless for this test set.

Although the H-factor and the R-factor are mathematically unrelated, they have the same purpose: to assess the quality of structures, either experimental or computed from modeling experiments, where quality refers to reflecting correctly the input data used to generate these structures. The H-factor mimics the R-factor as it provides a quality-index to follow in the process of building a model, the same way crystallographers monitor the R-factor/R-free indexes during structures refinements. We compared the H-factor results with the experimental R-factor and R-free on a randomly chosen subset of the PDB containing 445 structures with 6 or more identical chains solved by X-ray crystallography. Results are shown in Figure 5. The H-factor and R-factor are not linear correlated but it remains that "good" R-factors (below 30%) correspond to "good" H-factor values (below 45%). The H-factor checks the diversity of the set of models generated for a structure, as well as their similarities with the structures of domains that share the same function, as defined by pfam. High H-factor values may be caused by structures with disordered loops or remote structural neighbors in the PDB. It remains that, in agreement with what we observed for the NMR structures, the H-factor recognizes experimentally determined structures as being valid.

### Relationship between the H-factor and cRMS

The cRMS by itself is a reasonable quality indicator as long as its value remains low (say below 2 Å). It should be noted that it is an average value computed over the whole structure. As such, it is very sensitive to large structural fluctuations in disordered loops for example that can lead to large cRMS values even if the conserved domains are structurally very similar. It is well known for example that caution should be applied when using cRMS to assess the quality of a structural alignment. cRMS is implemented in score ***(3) ***of the H-factor to evaluate the heterogeneity amongst a set of models as it is directly related to both the choice of the template and the quality of the sequence alignment. However, the score ***(3) ***loses accuracy for cRMS values larger than 2 Å, which is not uncommon when a remote template is used. cRMS is also implemented in score ***(4) ***to quantity the modelling quality of specific individual domains by comparing them with corresponding domains in the PDB. This is a domain-based cRMS that does not take into account potential long loops between domains, making it more reliable. Taken together, the scores ***(3) ***and ***(4) ***alleviate most of the limitations of cRMS while retaining its major properties. The H-factor is therefore expected to be more reliable than a sole cRMS to judge the accuracy of a wide range of models, as seen in Table 3.

### Comparing the H-factor with ProSA, DOPE and QMEAN

**Figure 6:**
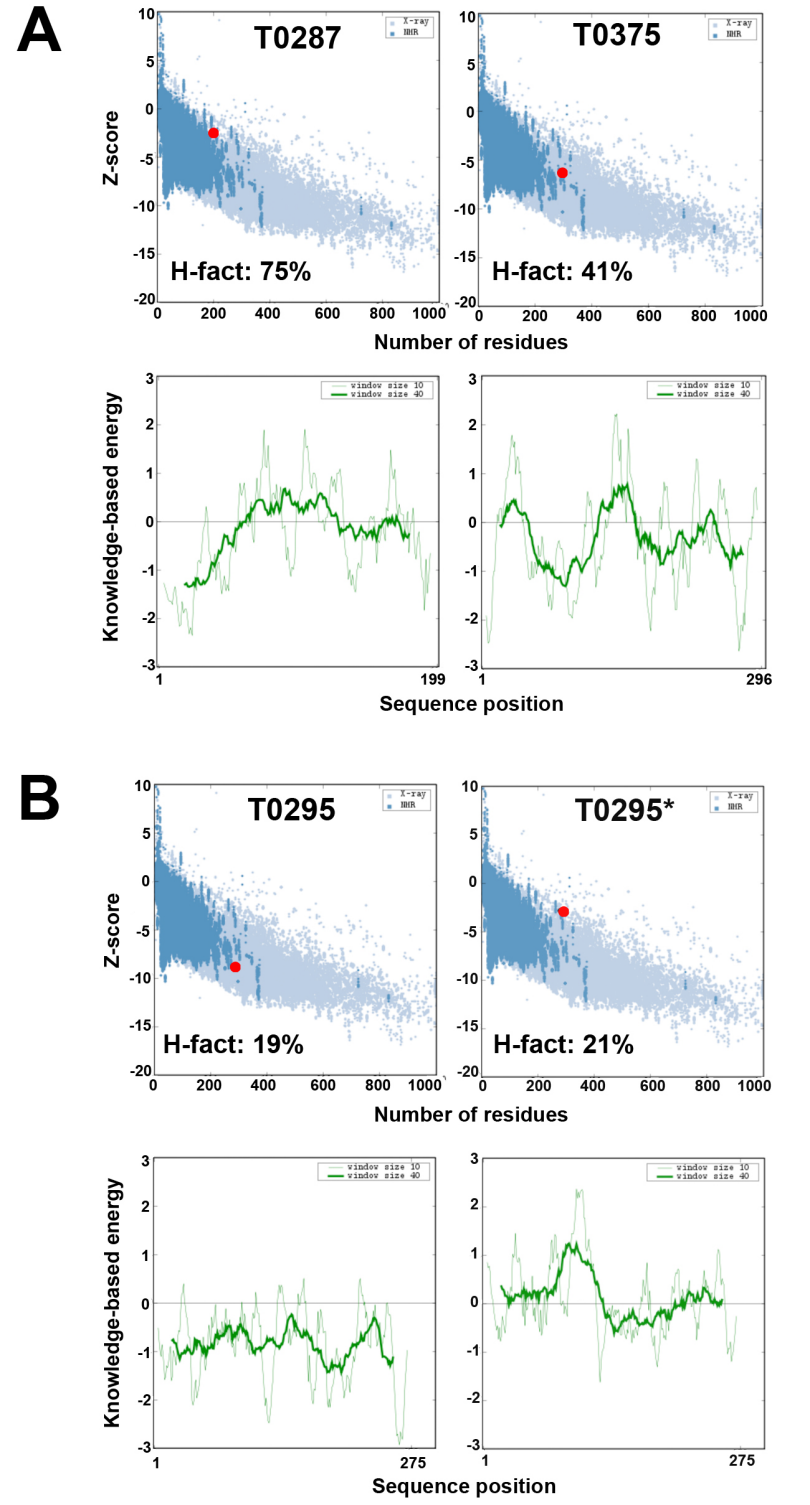
**Comparison between the H-Factor and ProSA**. ProSA-Web analysis of the average model generated for three CASP7 targets: **A**. T0287 and T0375. **B**. T0295 and T0295*. The test case T0295* corresponds to T0295 with a modified sequence alignment used to generate the models (see figure 2 for details). The H-factors for the same models are given for comparison.

The structural biology community as well as the protein structure modeling community have always been reasonably good at setting safeguards to estimate the validity of both experimental and computational models for protein structures. The first validation tests imposed on experimental structures focused on the stereochemistry of the molecules. It was found, however that this was by far not sufficient, as "good stereochemistry" can be misleading. A "clean" Ramachandran plot for example does not necessary prove that a given model is valid. Also, we observed that all the models listed in Table 1 have good stereochemistry, including the T0287 model that has a Cα backbone deviation of almost 6 Å from the actual experimental structure. Experimentalists have access to another essential set of validation tools that check the consistency of the structures with the experimental data that were used to derive them. Such tools include the R factors in X-ray crystallography and NMR. Models derived from modeling experiments are more difficult to verify, unless they include some external information. For example, the SWISS-model server provides a confidence score along with the models it built and this confidence score is based on the amount of structural information that supports each part of the model [38,90]. In general, however, validation of computer-generated models is based on comparison with known protein structures. The idea is that basic properties of protein structures can be inferred from the PDB, and translated into database-derived scoring functions. This is the main rational for the statistical potentials, also referred to as mean-field potentials that have become very popular validation tools [67,91,92]. For example, ProSA-web implement two statistical potentials: a pairwise potential based on Cß atoms and a surface term that models the protein-solvent interactions [93]. Here we compare the output of ProSA and H-factor analyses on the three CASP7 test cases given in table 1. The ProSA results are given if Figure 6. Both the H-factor and ProSA estimate that the models generated for T0295 are valid. When the modeling process is deliberately altered (test case T0295*, see above for details), again both ProSA and the H-factor analyses reveal a loss of quality. ProSA however considers the models generated for the targets T0287 and T0375 as statistically correct (i.e. within range of what is observed in native proteins), while we know that these models are not correct (see table 2). In addition, it is striking to see that the PDB structure 1US0, an ultra high-resolution crystal structure at 0.66Å resolution, has a ProSA Z-score (-12.27) statistically as correct as T0287 (data not shown).

The statistical potential Discrete Optimized Protein Energy (DOPE) is another measure of model quality that has been introduced in MODELLER-8 [94]. DOPE is a statistical potential with an improved reference state that accounts for the compact shape of native protein structures. The DOPE score is designed such that large, negative scores are usually indicators of good models. In their original study, Shi and Sali [94] found that the accuracy of DOPE to asses a homology model improves as the accuracy of the models improve. We observe a similar behaviour for targets T0295 and T0295* (Table 2). These two targets correspond to the same protein and it is therefore possible to compare the DOPE scores of their models. The model generates for T0295*, based on an incorrect alignment, has a much lower DOPE score (-24317) that the model generated with the correct alignment (T0295;-33940). Note that we cannot compare DOPE scores for proteins of different size, as these scores are not normalized. DOPE scores are therefore relative, and designed to pick a "good" model among poorer model. DOPE scores do not assess directly the quality of the model that is picked, i.e. if it is likely to be similar to the actual structure. The H-factor is a better indicator in that respect.

QMEAN, which stands for Qualitative Model Energy ANalysis, is a composite scoring function for homology models that describes the major geometrical aspects of protein structures (including a torsion angle potential over three consecutive amino acids, a secondary structure-specific residue-based statistical potential, a solvation potential for the burial of residues) as well as the agreement between the predicted and calculated secondary structure and solvent accessibility, respectively [95]. As such, it includes a term similar to the score ***(1) ***of the H-factor, as well as terms that assess different properties such as residue accessibility. The score QMEANnorm is a normalised version of the QMEAN score in which all terms are divided by the number of interactions/residue in order to avoid a size-bias of the score [95]. QMEANnorm scores vary between 0 and 1, with larger scores expected to correspond to better models. Unlike the DOPE score, both the H-factor and QMEANnorm scores allow for the comparison of proteins of different sizes. The QMEANscore is as effective as PROSA or DOPE for detecting errors in a model that result from errors in the sequence alignment between the template and target protein: T0295* has a QMEANnorm score of 0.196 while the score forT0295 is 0.735 (Table 2). Interestingly, T0295* (0.196) has a less favorable QMEANnorm score than the erroneous model generated for the CASP target T0287 (0.285) (see table 3). We have observed however that the QMEANnorm score is prone to fail: some of the erroneous models generated for the CASP target T0307 have QMEANscores of 0.4 to 0.6, i.e. they are evaluated to be almost as correct as the positive control T0295 (0.735). Unlike ProSA and QMEAN, the H-factor did detect that these models were to be considered with caution. Because it analyzes a set of models, we believe that the H-factor score is more robust as an absolute measure of the quality of a model. It lacks however the ability to discriminate among a set of models generated for the same target; PROSA and DOPE are better potentials for this specific task.

These results emphasize the essential differences in the nature of the ProSA, DOPE, QMEANnorm and H-factor scores. ProSA, DOPE and QMEAN check the quality of a model, independently of the context in which it was generated. The H-factor on the other hand checks the quality of a set of models with respect to a context that includes for example the sequence alignment assessed by the score ***(2)*. **The modeler however should use these differences to extend his/her assessment of the model his/she generates. We believe that ProSA, DOPE, QMEAN and H-Factor analyses are needed to provide a better overview of the quality of models derived by homology modeling.

### Current limitations and originalities of the H-factor

The H-factor is not the *panacea*, and does not provide a universal solution to the problem of asserting the quality of a model generated by homology modeling. Firstly, the H-factor has some technical limitations. Our current implementation does not take into account multiple templates, but rather only one single framework. The structural components included in the H-factor (i.e. scores ***(3) ***and ***(4)***) are based on the backbone of the models, and do not take into account sidechains and possible errors in their modeling. Second, the scoring function ***(3) ***of the H-factor measures the heterogeneity of a set of models generated with the same input. It means, that the H-factor cannot be computed on a singular model. In homology modeling the heterogeneity of models can be seen as a quality indicator and building only one single model is not recommended. Similarly to NMR structures where only one of the models can be chosen for analysis, the best model in homology modeling regime is chosen based on the MODELLER energy function for instance. Third, the H-factor does not include any external information. For example, if some biological data are available, such as the knowledge of the residues involved in the active site, or standard biophysical data such as melting temperature, or secondary structure content derived for circular dichroism, these data are currently not included in the H-factor analysis.

The R-factor is a measure of the agreement between the crystallographic model and the experimental X-ray diffraction data. Despite the lack of 'experimental' data to compare with, the modelling community has been searching for a similar indicator for homology modeling. Both QMEAN and the H-factor are designed to be 'absolute' indicators that assess the quality of homology models in a way that mimics the R-factor in X-ray crystallography. Both QMEAN and H-factor provide an easy-to-use estimate of the quality of models based on scoring functions assessing various aspects of the modelling process as well as the model itself [95].

*In vivo *macromolecular structures oscillate between numerous conformers, some more than others. While X-ray structures correspond to snapshots of a limited numbers of conformers, NMR structures tend to describe more accurately flexibility. Indeed, NMR "structures" are usually provided as a family of conformers that are meant to sample the conformational space accessible to the molecule of interest. In homology modeling on the other hand, the heterogeneity of models is a quality indicator. A good set of models will have a cRMS very close to their framework. Moreover, if errors are being made in the template choice or in the sequence alignment, then the models will be heterogeneous. The scoring function ***(3) ***is designed to quantify this assertion. It also means that the H-factor cannot be computed on a single model.

One of the originality of the H-factor is the scoring function ***(4)***. It has been designed to evaluate the biological relevance of the models by comparing the model conformations of all the functional domains in the protein considered with the existing sibling deposited in the Protein Data Bank.

We acknowledge that there is room for improvement. However, It remains that the H-factor we have introduced here is a first step in the direction of validating homology models for the biologists in addition to existing methods, as proved in the examples shown above.

## Conclusions

Homology modeling is slowly building up a record of success and can help structural biologists in many aspects. Models can serve as a bootstrap structure for both NMR and X-ray crystallography and thus help saving a huge amount of time. In X-ray crystallography for instance, many derivative dataset are often needed to solve the phase problem. Alternatively, an accurate bootstrap would be extremely handy for molecular replacement. The same applies for NMR. Protein modeling is also crucial for fitting low resolution electron microscopy maps or building accurate models using structural restraints gathered with small-angle X-ray scattering (SAXS) experiments. Models can be used at different level of details according to their accuracy. In the absence of experimental structures, they serve as starting points for modeling experiments, such as molecular dynamics studies, docking experiments and structure-based drug design. For instance, models of membrane proteins such as G-protein-coupled receptor (GPCR) are extensively used, as few structures are available for this protein family [96].

In this study, we proposed a modeling etiquette that hopefully will help make good use of models. We introduced the H-factor, a new indicator that assesses the quality of models generated by homology modeling, mimicking the R-factor in X-ray crystallography. The H-factor is able to detect backbone anomalies as well as give a feedback on the biological relevance of models. The H-factor evaluates the quality of a protein model within the context in which it is modelled and we believe it is an essential tool that needs to be used in addition to the other validation tools available.

## Note

To search for protein structures using any of the accession numbers mentioned in this article, please follow this link (http://www.rcsb.org/pdb/home/home.do).

## Authors' contributions

EDL & PK conceived and designed the experiments. EDL performed the experiments. EDL & PK analyzed the data. EDL & PK wrote the paper.

## Supplementary Material

Additional file 1 **Sequence alignments used to build models for CASP7 targets used to test the H-Factor**. Sequence alignments for **(1) **T0287; **(2) **T0295 and **(3) **T0375 respectively. Click here for file

Additional file 2 **A simplified operating manual for the H-Factor**. Operating manual for the online H-Factor server. Click here for file
