# Supplementary material for: A quality metric for homology modeling: the H-factor
Source: BMC Bioinformatics. 2011 Feb 4;12:48. doi: 10.1186/1471-2105-12-48 (PMC3213331; doi:10.1186/1471-2105-12-48)
Supplement: Additional file 1 — Sequence alignments used to build models for CASP7 targets used to test the H-Factor. Sequence alignments for (1) T0287; (2) T0295 and (3) T0375 respectively. [file 1471-2105-12-48-S1.DOC]

**Additional files**

**Additional file 1**

**(1)** T0287

1V55 FINRWLFSTNHKDIGTLYLLFGAWAGMVGTALSLLIRAELGQPGTLLGDDQIYNVVVTAH 60

T0287 ---------MSNNMRKLFSMIADSKDKKEKLIESLQENELLNTD---EKKKIIDQIKTMH 48

::: .*: ::. . . :. * . ** :.. ..:* : : * *

1V55 AFVMIFFMVMPIMIGGFGNWLVPLMIGAPDMAFPRMNNMSFWLLPPSFLLLLASSMVEAG 120

T0287 DFFKQMHTNKGALDKVLRNYMKDYRAVIKSIGVDKFKKVYRLLESETMELLHA------- 101

*. :. : : *:: .:.. ::::: * . :: ** *

1V55 AGTGWTVYPPLAGNLAHAGASVDLTIFSLHLAGVSSILGAINFITTIINMKPPAMSQYQT 180

T0287 ----------IAENPNFLFSKFDRSILGIFLP---------------------------- 123

:* * . :..* :*:.:.*.

1V55 PLFVWSVMITAVLLLLSLPVLAAGITMLLTDRNLNTTFFDPAGGGDPILYQHLFWFFGHP 240

T0287 ------FFSKPIMFKMSIREMDSQIELYGTN----------------------------- 148

.: ..::: :*: : : * : *:

1V55 EVYILILPGFGMISHIVTYYSGKKEPFGYMGMVWAMMSIGFLGFIVWAHHMFTVGMDVDT 300

T0287 ---LPLLKLFVMTDEEVNFYANLKTIEQYNDYVRDLL------------------MKFDL 187

: :* * * .. *.:*:. * * . * :: *..*

1V55 RAYFTSATMIIAIPTGVKVFSWLATLHGGNIKWSPAMMWALGFIFLFTVGGLTGIVLANS 360

T0287 EKYMKEKGVQNA------------------------------------------------ 199

. *:.. : *

1V55 SLDIVLHDTYYVVAHFHYVLSMGAVFAIMGGFVHWFPLFSGYTLNDTWAKIHFAIMFVGV 420

T0287 ------------------------------------------------------------

1V55 NMTFFPQHFLGLSGMPRRYSDYPDAYTMWNTISSMGSFISLTAVMLMVFIIWEAFASKRE 480

T0287 ------------------------------------------------------------

1V55 VLTVDLTTTNLEWLNGCPPPYHTFEEPTYVNLK 513

T0287 ---------------------------------

**(2)** T0295

1zq9 QHILKNPLIINSIIDKAALRPTDVVLEVGPGTGNMTVKLLEKAKKVVACELDPRLVAELH 60

T0295 -HLLKNPGILDKIIYAAKIKSSDIVLEIGCGTGNLTVKLLPLAKKVITIDIDSRMISEVK 59

*:**** *::.** * ::.:*:***:* ****:***** ****:: ::*.*:::*::

1zq9 KRVQGTPVASKLQVLVGDVLKTDLPFFDTCVANLPYQISSPFVFKLLLHRPFFRCAILMF 120

T0295 KRCLYEGYN-NLEVYEGDAIKTVFPKFDVCTANIPYKISSPLIFKLISHRPLFKCAVLMF 118

** :*:* **.:** :* **.*.**:**:****::***: ***:*:**:***

1zq9 QREFALRLVAKPGDKLYCRLSINTQLLARVDHLMKVGKNNFRPPPKVESSVVRIEPKNPP 180

T0295 QKEFAERMLANVGDSNYSRLTINVKLFCKVTKVCNVNRSSFNPPPKVDSVIVKLIPKESS 178

*:*** *::*: **. *.**:**.:*:.:* :: :*.:..*.*****:* :*:: **:..

1zq9 PPINFQEWDGLVRITFVRKNKTLSAAFKSSAVQQLLEKNYRIHCSVHNIIIPEDFSIADK 240

T0295 FLTNFDEWDNLLRICFSRKRKTLHAIFKRNAVLNMLEHNYKNWCTLN-KQVPVNFPFKKY 237

**:***.*:** * **.*** * ** .** ::**:**: *::: :* :*.: .

1zq9 IQQILTSTGFSDKRARSMDIDDFIRLLHGFNAEGIHFS 278

T0295 CLDVLEHLDMCEKRSINLDENDFLKLLLEFNKKGIHFF 275

::* .:.:**: .:* :**::** ** :****

**(3)** T0375

1RKD ----AGSLVVLGSINADHILNLQSFPTPGETVTGNHYQVAFGGKGANQAVAAGRSGAN-I

1VM7 ---MFLVISVVGSSNIDIVLKVDHFTKPGETQKAIEMNVFPGGKGANQAVTVAKIGEKGC

2FV7 ----VAAVVVVGSCMTDLVSLTSRLPKTGETIHGHKFFIGFGGKGANQCVQAARLGAM-T

1V1A ----MLEVVTAGEPLVALVP-----QEPGHLRGKRLLEVYVGGAEVNVAVALARLGVK-V

2DCN -----AKLITLGEILIEFNA-----LSPGPLRHVSYFEKHVAGSEANYCVAFIKQGNE-C

2AFB HHHHHMKVVTFGEIMLRLSP-----PDHKRIFQTDSFDVTYGGAEANVAAFLAQMGLD-A

T0375 ----GSQILCVGLVVLDVISLVDKYPKEDSEIRCLSQRWQRGGNASNSCTILSLLGAP-C

: * .* * .. *

1RKD AFIACTGDDSIGESVRQQLATDNIDITPVSVIKGESTGVALIFVN----GEGENVIGIHA

1VM7 RFVTCIGNDDYSDLLIEN--YEKLGITGYIRVS-LPTGRAFIEVD----KTGQNRIIIFP

2FV7 SMVCKVGKDSFGNDYIENLKQNDISTEFTYQTKDAATGTASIIVN----NEGQNIIVIVA

1V1A GFVGRVGEDELGAMVEERLRAEGVDLTHFRRAPGFTG---LYLREYLPLGQGRVFYYRKG

2DCN GIIAKVGDDEFGYNAIEWLRGQGVDVSHMKIDPSAPTGIFFIQRHYPVPLKSESIYYRKG

2AFB YFVTKLPNNPLGDAAAGHLRKFGVKTDYIARGGNRIGIYFLEIGASQ--RPSKVVYDRAH

T0375 AFMGSMAPGHVADFVLDDLRRYSVDLRYTVFQTTGSVPIATVIIN---EASGSRTILYYD

:: . . : .

1RKD GANAALSPALVEAQR-----ERIANASALLMQLESPLESVMAAAKIAHQNKTIVALNP--

1VM7 GANAELKKELIDWN-------TLSESDILLLQNEIPFETTLECAKRFNG---IVIFDP--

2FV7 GANLLLNTEDLRAAA-----NVISRAKVMVCQLEITPATSLEALTMARRSGVKTLFNP--

1V1A SAGSALAPGAFDPDYLEG-VRFLHLSGITPALSPEARAFSLWAMEEAKRRGVRVSLDVNY

2DCN SAGSKLSPEDVDEEYVKS-ADLVHSSGITLAISSTAKEAVYKAFEIASNR----SFDTNI

2AFB SAISEAKREDFDWEKILDGARWFHFSGITPPLGKELPLILEDALKVANEKGVTVSCDLNY

T0375 RSLPDVSATDFEKVDLTQFKWIHIEGRNASEQVKMLQRIDAHNTRQPPEQKIRVSVEVE-

: . . :

1RKD ---APAR-ELPDELLALVDIITPNETEAEKLTGIRVENDE--------DAAKAAQVLHEK

1VM7 ---APAQ-GINEEIFQYLDYLTPNEKEIEALSKDFFGEFL--------TVEKAAEKFLEL

2FV7 ---APAIADLDPQFYTLSDVFCCNESEAEILTGLTVGSAA--------DAGEAALVLLKR

1V1A RQTLWSPEEARGFLERALPGVDLLFLSEE--EAELLFG----------RVEEALRALS--

2DCN RLKLWSAEEAKREILKLLSKFHLKFLITDTDDSKIILGES--------DPDKAAKAFSDY

2AFB RARLWTKEEAQKVMIPFMEYVDVLIANEEDIEKVLGISVEGLNREAYAKIAEEVTRKYNF

T0375 --------KPREELFQLFGYGDVVFVSKDVAKHLGFQS-----------AEEALRGLYGR

: : :

1RKD GIRTVLITLGSRG------VWASVNGEGQRVPGFR-VQAVDTIAAGDTFNGALITALL--

1VM7 GVKNVIVKLGDKG------VLLVNKNEKKHFPTFK-VKAVDTTAAGDVFNGAFAVALS--

2FV7 GCQVVIITLGAEGCV----VLSQTEPEPKHIPTEK-VKAVDTTGAGDSFVGALAFYLAYY

1V1A -APEVVLKRGAKG------AWAFVDGRRVEGSAFA-VEAVDPVGAGDAFAAGYLAGAV--

2DCN -AEIIVMKLGPKG------AIVYYDGKKYYSSGYQ-VPVEDVTGAGDALGGTFLSLYY--

2AFB KTVGITLRESISATVNYWSVMVFENGQPHFSNRYE-IHIVDRVGAGDSFAGALIYGSL--

T0375 VRKGAVLVCAWAEEG---ADALGPDGKLLHSDAFPPPRVVDTLGAGDTFNASVIFSLS--

: . . . * .*** : .

1RKD EEKPLPEAIRFAHAAAAIAVTRKGAQPSVPWREEIDAFLDRQR

1VM7 EGKNPEEAVIFGTAAAAISVTRLGAQSSIPAREEVEAFLKNL-

2FV7 PNLSLEDMLNRSNFIAAVSVQAAGTQSSYPYKKDLPLTLF---

1V1A WGLPVEERLRLANLLGASVAASRGDHEGAPYREDLEVLLK---

2DCN KGFEMEKALDYAIVASTLNVMIRGDQENLPTTKDIETFLREM-

2AFB MGFDSQKKAEFAAAASCLKHTIPG-DFVVLSIEEIEKLASG--

T0375 QGRSVQEALRFGCQVAGKKCGLQGFDGIV--------------

. . . * .
