# Supplementary material for: A quality metric for homology modeling: the H-factor
Source: BMC Bioinformatics. 2011 Feb 4;12:48. doi: 10.1186/1471-2105-12-48 (PMC3213331; doi:10.1186/1471-2105-12-48)
Supplement: Additional file 2 — A simplified operating manual for the H-Factor. Operating manual for the online H-Factor server. [file 1471-2105-12-48-S2.DOC]

**Additional file 2**

A beta version of the H-factor is accessible online at <http://koehllab.genomecenter.ucdavis.edu/toolkit/h-factor>

The H-factor is computed only for a set of models build with the same input. The following elements are required for the H-factor computation:

1. The target protein sequence (the model sequence) in FASTA format (one letter format).
2. The template structure in PDB format.
3. The protein sequence alignment between the model and the template in PIR or CLUSTALW format
4. The model structures (up in 25 models) in PDB format.

Once all the data have been uploaded, the server will compute the H-factor. It may takes up to 30 minutes to return a result depending on the server load. You will be notified by email upon completion. The results can then be downloaded; they remain accessible on the server for 24 hours.
